# Continuum of maternal and newborn health in Sierra Leone: a 2019 national survey

**DOI:** 10.1186/s13690-022-00946-8

**Published:** 2022-08-09

**Authors:** Quraish Sserwanja, Linet M. Mutisya, Lilian Nuwabaine, Kassim Kamara, Ronald K. Mutebi, Milton W. Musaba

**Affiliations:** 1 Programmes Department, GOAL Global, Arkaweet Block 65 House No. 227, Khartoum, Sudan; 2 Maternal and Child Health Project, Swedish Organization for Global Health, Mayuge, Uganda; 3 School of Nursing and Midwifery, Aga Khan University, Kampala, Uganda; 4 National Disease Surveillance Programme, Ministry of Health and Sanitation, Free town, Sierra Leone; 5 Clinical Epidemiology Unit, Makerere University, Kampala, Uganda; 6 Department of Internal Medicine, Mengo Hospital, Kampala, Uganda; 7 Department of Obstetrics and Gynaecology, Mbale Regional Referral and Teaching Hospital, Mbale, Uganda; 8 Department of Obstetrics and Gynaecology, Busitema University, Mbale, Uganda

**Keywords:** Continuum of care, Sierra Leone, DHS, Maternal care

## Abstract

**Introduction:**

Globally, Sierra Leone has some of the worst maternal and child health indicators. The situation is worsened by a dearth of evidence about the level of continuum of care, an evidence-based intervention aimed at reducing maternal and perinatal morbidity and mortality. Hence this study aimed to assess the level of and factors associated with continuum of maternal and newborn care in Sierra Leone.

**Method:**

This study analyzed secondary data from the 2019 Sierra Leone Demographic Health Survey. Analysis was restricted to women who had a live birth in the 5 years preceding the survey (*n* = 7326). Complete continuum of care was considered when a woman reported having had at least eight antenatal care contacts, skilled birth attendance and mother and baby had at least one postnatal check-up. Bi-variable and multivariable logistic regression were performed using the statistical package for the social sciences software version 25.

**Results:**

Only 17.9% (95% CI: 17.4–19.1) of the women utilized complete continuum of care for maternal and newborn health services in Sierra Leone. About 22% (95% CI: 21.3–23.1) utilized 8 or more antenatal care contacts, 88% (95% CI: 87.9–89.4) had skilled birth attendance while 90.7% (95% CI: 90.2–91.5) and 90.4% (95% CI: 89.9–91.2) of mothers and neonates utilized postnatal care respectively. Having started antenatal care within the first trimester (aOR 1.71, 95% CI: 1.46–2.00), being resident in the Southern region (aOR 1.85, 95% CI: 1.23–2.80), belonging to richer wealth quintile (aOR 1.76, 95% CI: 1.27–2.44), using internet (aOR 1.49, 95% CI: 1.12–1.98) and having no big problems seeking permission to access healthcare (aOR 1.34, 95% CI: 1.06–1.69) were significantly associated with utilization of continuum of care.

**Conclusion:**

The overall completion of continuum of maternal care is low, with ANC being the lowest utilized component of continuum of care. These findings call for urgent attention for maternal health stakeholders to develop and implement tailored interventions prioritizing women empowerment, access to affordable internet services, timely initiation of ANC contacts, women in developed regions such as the Western and those from poor households.

## Introduction

Globally, Maternal and child health (MCH) is still a key public health priority because many countries risk missing the sustainable development goal (SDG) 3 targets of reducing maternal mortality ratio (MMR) and neonatal mortality ratio (NMR) to less than 70 deaths per 100,000 live and 12 per 1000 live births respectively by 2030 [1, 2]. Many countries across the globe continue to grapple with high maternal and perinatal mortality rates [2]. Estimates indicate that over 810 women die due to maternal related complications every day, with over 94% of these deaths occurring in low and middle income countries [3]. Sub-Saharan Africa alone accounts for approximately 67% of the maternal deaths with over 462 deaths per 100,000 live births [4]. Over 27·8 million children die globally in their first 28 days of life, and majority of these occur in sub Saharan Africa [5].

Even within sub-Saharan Africa (SSA), major disparities exist with countries like Zambia having a MMR of 252 deaths per 100,000 live births [6, 7] while Sierra Leone not only remains one of the poorest countries, it also reports some of the worst MCH indicators globally [8–10]. For instance, the MMR stands at 717 deaths per 100,000 live births, while the neonatal mortality ratio (NMR) is 31 deaths per 1000 live births respectively [11, 12].

The World Health Organization has recommended a number of interventions and strategies to strengthen the continuum of care and these have been adopted by many countries including Sierra Leone [5, 13]. These include integrated and uninterrupted antenatal care (ANC), skill birth attendance (SBA), and postnatal care (PNC) packages as critical strategies in reducing maternal and neonatal mortality and morbidity [13]. Timely ANC contacts are meant to effect birth preparedness, enable identification and treatment of illness during pregnancy [14–16]. Having skilled birth attendance from qualified and experienced professionals assures safe delivery with the necessary drugs and supplies for effective prevention, management of emergencies and also for referral in case of any obstetric complications [6, 17]. Overall, completion of the continuum of care follows a pathway from pregnancy through delivery to the postpartum period, where each step adds value to ensure better health outcomes for mothers and newborns through timely identification of complications, which would contribute in maternal and neonatal mortality reduction [18–20].

The concept of maternal and newborn continuum of care is grounded in the assumption that well-being and health of women and newborns should be closely linked and managed in a unified way [21]. In addition to saving lives of mothers, completion of the continuum can save up to 160,000 newborn lives by maximizing ANC coverage, 390,000 additional newborn lives through high coverage of skilled childbirth care, and save 310,000 lives through postnatal care [22]. Early initiation and completion of the continuum could greatly help in solving challenges and improve the health and survival of women, newborns, and children worldwide [6, 17]. However, studies across Africa continue to reveal very low completion rates for the continuum of care [6, 20, 23, 24]. These low rates in the trends have been associated with socio-economic factors like educational status, residence, distance from the health facility, woman’s decision making power, early initiation of ANC, wealth index, media access, skills of health workers, health system supports, and presence of delivery fees [4, 6, 22, 25]. These continue to affect the continuity of the continuum and pose setbacks to the fight against maternal and neonatal mortality especially in low- and middle-income countries.

Although the government of Sierra Leone has come up with policies and interventions aimed at improving maternal and child health services such as the 2010 Free Health Care Initiative (FHCI) and the national reproductive, maternal, neonatal, child and adolescent health (RMNCAH) strategy (2017–2021), not much improvement has been registered regarding impact level MCH indicators since the country has one of the worst MMR globally [26, 27]. The high MMR despite the 2017–2021 RMNCAH strategy prioritizing continuum of care approach, evidence based interventions and aligning ANC interventions to the 2016 WHO ANC model that recommends a minimum of 8 ANC contacts could be partially attributed to limited availability of skilled health workers, increasing workload and inadequate medical supplies and limited data on topics such as completion of continuum of care [12, 27–29]. Analysis of the level of completion of the continuum of care can provide data that can be used to formulate evidence based policies aimed at increasing maternal health services utilization. Therefore, this study is aimed at assessing the level and determinants of completion of the continuum of care for maternal and newborn health in Sierra Leone, using secondary data from the most recent national demographic health survey of 2019. This is the fore most study that analyses the continuum of care for maternal and newborn health considering the latest WHO ANC guidelines that were adopted in the Sierra Leone 2017–2021 RMNCAH strategy.

## Methods

### Study design and sampling methods

The Sierra Leone Demographic and Health Surveys (SLDHS) are cross-sectional surveys that are periodically conducted to obtain information on demographic, health and nutritional indicators of women of reproductive age (15–49 years), men (15 to 54 years) and children. This SLDHS was conducted over a 4 month period between 15th May 2019 and 31st August 2019 [11]. This national survey used stratified, two-stage cluster sampling design to obtain a representative sample of 13,872 households [11]. Weighted data was used to account for the unequal probability sampling in different strata. A detailed explanation of the sampling process is available elsewhere [11]. Women aged 15–49 years who were either permanent residents or visitors who had stayed in the selected households the night before the survey were eligible for interviews with a total of 15,574 women who were interviewed. Secondary analysis included women aged 15 to 49 years who had a live birth within 5 years preceding the survey (with the most recent birth being considered) and were either permanent residents or slept in the selected household the night preceding the survey. Out of the total weighted sample of 15,574 women in the data set, only 7326 had given birth within 5 years preceding the survey (as shown in Table 1). Of the 7326 women, 112 (1.5%) women had missing data on ANC initiation timing leading to a total of 7214 women who were considered for logistic regression analysis.

**Table 1:** Socio-demographic characteristics of women in Sierra Leone as per the 2019 SLDHS

Characteristics	***N*** = 7326	%
**Age**		
15 to 19	598	8.2
20 to 34	4830	65.9
35 to 49	1898	25.9
**Residence**		
Urban	2795	38.1
Rural	4531	61.9
**Region**		
Western	1479	20.2
Eastern	1542	21.0
Northwestern	1380	18.8
Northern	1433	19.6
Southern	1492	20.4
**Religion**		
Islam	5766	78.7
Christianity and others	1560	21.3
**Sex household head**		
Male	5520	75.3
Female	1806	24.7
**Household Size**		
7 and above	3319	45.3
Less than 7	4007	54.7
**Working status**		
Not working	1683	23.0
Working	5643	77.0
**Marital status**		
Not married	1329	18.1
Married	5997	81.9
**Education Level**		
No Education	3857	52.7
Primary Education	1033	14.1
Secondary Education	2214	30.2
Tertiary	221	3.0
**Wealth Index**		
Poorest	1587	21.7
Poorer	1551	21.1
Middle	1487	20.3
Richer	1441	19.7
Richest	1259	17.2
**ANC initiation timing**^**a**^		
First trimester	3214	44.6
After first trimester	4000	55.4
**Parity**		
1	1989	27.1
2–4	4015	54.8
5 and above	1323	18.1
**Exposure to newspapers**		
No	6921	94.5
Yes	405	5.5
**Exposure to Radio**		
No	4224	57.7
Yes	3102	42.3
**Exposure to TV**		
No	5579	76.2
Yes	1747	23.8
**Internet use**		
No	6586	89.9
Yes	740	10.1
**Permission to access healthcare**		
Big problem	1827	24.9
Not big problem	5499	75.1
**Distance to health facility**		
Big problem	3454	47.1
Not big problem	3872	52.9

^a^_=_ missing 112 (1.5%) respondents

### Variables

#### Outcome variables

Complete continuum of maternal and newborn healthcare was the outcome variable and was constructed into a binary variable with complete coded as 1 and incomplete coded as 0. Complete continuum of maternal and newborn healthcare was considered when a woman reported having had all the three conditions/states:

1. Had at least eight ANC contacts during the most recent childbirth.
2. Skilled birth attendance
3. Mother and baby had at least one postnatal check-up within 6 weeks after childbirth

#### Independent variables

Eighteen independent variables were categorized into women and household characteristics, and were chosen basing on previous studies [20, 21, 30] and availability in the SLDHS database.

#### Household characteristics

Wealth index of household (categorized into quintiles: richest, richer, middle poorer and poorest), type of residence (urban and rural), and region that included the official five regions in the SLDHS (western, eastern, southern, northwestern and northern), household size (was originally a continuous variable and we categorized it into; less than 7 and, 7 and above) and sex of household head (male and female) Wealth index is a measure of relative household economic status and was calculated by DHS from information on household asset ownership using principal component analysis [11, 31].

#### Women’s characteristics

Age (was originally a continuous variable and we categorized it into; 15–24, 25–34, and 35–49 years), level of education (no education, primary, secondary, and tertiary), exposure to newspapers/magazines, internet, radio and TV (yes and no), parity (1, 2–4 and 5 and above), ANC initiation timing (first trimester and after first trimester), marital status (married and not married), working status (working and not working) and decision making for seeking healthcare (involved and not involved). Religion was categorized as Islam and Christianity and others while problems seeking permission and distance to health facility were categorized as big problem and no big problem. In the questionnaire, seeking permission to access healthcare and distance to health facility had three original responses: no problem, no big problem and big problem. However, none of the study participants reported no problem hence we only had two responses.

### Statistical analysis

In order to account for the multi-stage cluster study design, complex sample package of SPSS (version 25.0) statistical software was used. Analysis was carried out based on the weighted count to account for the unequal probability sampling in different strata and to ensure representativeness of the survey results at the national and regional level.

Before multivariable logistic regression analysis, cross tabulation was done and then independent variables were assessed for their association with CoC utilisation using bivariable logistic regression analysis and we presented the crude odds ratio (cOR), 95% confidence interval (CI) and *p*-values. Independent variables associated with CoC utilisation from literature and those with a *p*-value ≤0.25 at the bivariable level, and not strongly collinear (considered variance inflation factor less than 3) with other independent variables were considered for multivariable logistic regression to assess the independent effect of each variable on the CoC utilisation. Adjusted odds ratios (aOR), 95% confidence intervals (CI) and *p*-values were calculated with statistical significance level set at *p*-value < 0.05. Sensitivity analysis was done considering the old WHO recommendations of at least 4 ANC visits.

## Results

A total of 7326 women were included in the analysis (Table 1). Of these, 734 (10.0%) (95% CI: 9.6–11.0) had complete continuum of care (further details in Table 2). Majority of the women had less than eight ANC contacts (78.0%), had had skilled birth attendance (88.3%) and had a postnatal check after delivery (90.7%). Most women resided in rural areas, belonged to Islam, had no education, resided in male headed households, were married, working and aged between 20 and 34 years. Most women had limited exposure to mass media with over 89.9% not having access to internet. The mean age and household size were 28.97 ± 7.25 years and 6.93 ± 3.45 members respectively.

**Table 2:** Utilisation of the different components of continuum of care

Service	Frequency ***N*** = 7326	%	95% CI
**8 or more ANC contacts**	1610	22.0	21.3–23.1
**4 or more ANC contacts**	5769	78.8	77.9–79.7
**Skilled birth attendance**	6468	88.3	87.9–89.4
**Postnatal Care**			
Maternal PNC at health facility discharge^a^	5706	92.3	91.7–93.0
Maternal PNC after discharge from health facility	2715	37.1	35.9–38.1
Maternal PNC (at least one of the above)	6646	90.7	90.2–91.5
Neonatal PNC at health facility discharge^a^	5735	92.8	92.1–93.4
Neonatal PNC after discharge from health facility	3329	45.5	44.6–46.8
Neonatal PNC (at least one of the above)	6625	90.4	89.9–91.2
PNC for both mother and neonate	6274	85.6	85.0–86.6
**Continuum of care**	1311	17.9	17.4–19.1

^a^Misssing 1143

### Factors associated with CoC utilisation

After adjusting for other variables, factors that were significantly associated with CoC utilisation were; having started ANC within first trimester (aOR 1.71, 95% CI: 1.46–2.00), belonging to the Southern region (aOR 1.85, 95% CI: 1.23–2.80), belonging to richer wealth quintile (aOR 1.76, 95% CI: 1.27–2.44), using internet (aOR 1.49, 95% CI: 1.12–1.98) and having no big problems seeking permission to access healthcare (aOR 1.34, 95% CI: 1.06–1.69) as shown in Table 3.

**Table 3:** Factors associated with CoC utilisation in Sierra Leone as per the 2019 SLDHS

Characteristics	Cross tabulation ***N*** = 7326			Logistic regression ***N*** = 7214		
	No COC n (%)	Yes, CoC n (%)	***P***-value	Crude model cOR (95% CI)	***P***-value	Adjusted model aOR (95% CI)
**Visited by fieldworker**			0.403		0.403	
No	4173 (69.4)	886 (67.6)		1		–
Yes	1842 (30.6)	425 (32.4)		1.09 (0.89–1.32)		
**ANC timing**^**a**^			< 0.001		< 0.001	
Above first trimester	2480 (42.0)	733 (55.9)		1		1
Within first trimester	3422 (58.0)	578 (44.1)		**1.75 (1.50–2.04)**		**1.71 (1.46–2.00)**
**Age**			0.735		0.771	
35 to 49	1548 (25.7)	350 (26.7)		1		–
20 to 34	3971 (66.0)	860 (65.6)		0.96 (0.83–1.11)	0.572	
15 to 19	496 (8.3)	101 (7.7)		0.90 (0.67–1.22)	0.504	
**Residence**			0.150		0.151	
Rural	3762 (62.5)	769 (58.7)		1		1
Urban	2253 (37.5)	542 (41.3)		1.17 (0.94–1.47)		0.99 (0.70–1.41)
**Region**			0.003		0.001	
Western	1227 (20.4)	252 (19.2)		1		1
Southern	1159 (19.3)	333 (25.4)		**1.40 (1.01–1.96)**	0.048	**1.85 (1.23–2.80)**
Northwestern	1167 (19.4)	213 (16.2)		0.89 (0.63–1.27)	0.520	1.16 (0.74–1.82)
Northern	1237 (20.6)	197 (15.0)		0.78 (0.53–1.13)	0.185	0.93 (0.61–1.44)
Eastern	1225 (20.3)	316 (24.1)		1.26 (0.87–1.83)	0.224	1.52 (0.99–2.33)
**Religion**			0.038		0.038	
Islam	4773 (79.3)	994 (75.8)		1		1
Christianity and others	1242 (20.7)	317 (24.2)		1.23 (1.01–1.49)		1.07 (0.87–1.32)
**Sex household head**			0.180		0.180	
Male	4557 (75.8)	963 (73.5)		1		1
Female	1458 (24.2)	348 (26.5)		1.13 (0.95–1.35)		1.06 (0.87–1.28)
**Household Size**			0.125		0.125	
7 and above	2755 (45.8)	564 (43.0)		1		1
Less than 7	3260 (54.2)	747 (57.0)		1.12 (0.97–1.30)		1.11 (0.95–1.29)
**Working status**			0.372		0.373	
Not working	1396 (23.2)	287 (21.9)		1		–
Working	4619 (76.8)	1024 (78.1)		1.08 (0.91–1.28)		
**Marital status**			0.214		0.215	
Not married	1073 (17.8)	256 (19.5)		1		1
Married	4942 (82.2)	1055 (80.5)		0.90 (0.76–1.07)		1.05 (0.87–1.26)
**Education Level**			< 0.001		< 0.001	
No Education	3226 (53.6)	632 (48.2)		1		1
Primary Education	829 (13.8)	204 (15.6)		1.26 (1.03–1.54)	0.027	1.13 (0.91–1.39)
Secondary Education	1801 (29.9)	413 (31.5)		1.17 (0.99–1.39)	0.066	0.93 (0.77–1.13)
Tertiary	159 (2.6)	62 (4.7)		**1.98 (1.43–2.75)**	< 0.001	1.26 (0.84–1.90)
**Wealth Index**			0.004		0.003	
Poorest	1358 (22.6)	229 (17.5)		1		1
Poorer	1288 (21.4)	263 (20.1)		1.21 (0.98–1.50)	0.082	**1.28 (1.02–1.60)**
Middle	1206 (20.0)	281 (21.4)		1.38 (1.12–1.71)	0.003	**1.45 (1.17–1.79)**
Richer	1127 (18.7)	315 (24.0)		**1.66 (1.27–2.16)**	< 0.001	**1.76 (1.27–2.44)**
Richest	1036 (17.3)	223 (17.0)		1.27 (0.93–1.74)	0.134	1.35 (0.86–2.10)
**Parity**			0.073		0.048	
5 and above	1118 (18.6)	204 (15.6)		1		1
2–4	3282 (54.6)	733 (55.9)		1.22 (1.02–1.46)	0.028	1.16 (0.95–1.41)
1	1615 (27.2)	374 (28.5)		1.27 (1.03–1.56)	0.025	1.17 (0.92–1.49)
**Newspapers exposure**			0.143		0.143	
No	5696 (94.7)	1225 (93.4)		1		1
Yes	319 (5.3)	86 (6.6)		1.25 (0.93–1.68)		0.98 (0.72–1.34)
**Exposure to Radio**			0.091		0.091	
No	3506 (58.3)	718 (54.7)		1		1
Yes	2509 (41.7)	593 (45.3)		1.16 (0.98–1.37)		1.05 (0.88–1.25)
**Exposure to TV**			0.391		0.391	
No	4562 (75.8)	1017 (77.6)		1		–
Yes	1453 (24.2)	294 (22.4)		0.90 (0.73–1.13)		
**Internet use**			< 0.001		< 0.001	
No	5461 (90.8)	1125 (85.8)		1		1
Yes	554 (9.2)	186 (14.2)		**1.63 (1.28–2.08)**		**1.49 (1.12–1.98)**
**Permission to access healthcare**			0.020		0.020	
Big problem	1549 (25.8)	277 (21.1)		1		1
Not big problem	4466 (74.2)	1034 (78.9)		**1.29 (1.04–1.61)**		**1.34 (1.06–1.69)**
**Distance to health facility**			0.802		0.802	
Big problem	2829 (47.0)	625 (47.7)		1		–
Not big problem	3186 (53.0)	686 (52.3)		0.98 (0.80–1.19)		

^a^_=_ missing 112 (1.5%) respondents, **Bold**: significant at < 0.05

In the sensitivity analysis (Table 4), 62.9% of the women completed the CoC. Initiation of ANC in the first trimester, belonging to all the other regions except the western, having tertiary education, listening to radio and belonging to poorer, middle, richer and richest wealth quintiles was associated with more odds of completing CoC compared to initiating ANC after first trimester, belonging to the western region, having no education, not listening to radio and belonging to the poorest wealth quintile respectively. In comparison to the primary analysis that considered 8 ANC contacts, only timing of ANC initiation, region and wealth index were the significant factors in both analyses. Level of education and listening to radio were only significant in the sensitivity analysis while problems with distance to health facility and permission seeking care were only significant in the primary analysis.

**Table 4:** Factors associated with CoC utilization as per the 2019 SLDHS considering 4 ANC contacts

Characteristics	Cross tabulation ***N*** = 7326			Logistic regression ***N*** = 7214		
	No COC n (%)	Yes, CoC n (%)	***P***-value	Crude model cOR (95% CI)	***P***-value	Adjusted model aOR (95% CI)
**Visited by fieldworker**			0.106		0.106	
No	1927 (70.9)	3133 (68.0)		1		1
Yes	790 (29.1)	1476 (32.0)		1.15 (0.97–1.36)		1.08 (0.92–1.28)
**ANC timing**^**a**^			< 0.001		< 0.001	
Above first trimester	1589 (61.0)	2411 (52.3)		1		1
Within first trimester	1015 (39.0)	2198 (47.7)		**1.43 (1.24–1.64)**		**1.33 (1.16–1.52)**
**Age**			0.027		0.020	
35 to 49	742 (27.3)	1157 (25.1)		1		1
20 to 34	1784 (65.7)	3046 (66.1)		**1.36 (1.10–1.69)**		1.02 (0.87–1.20)
15 to 19	191 (7.0)	407 (8.8)		**1.56 (1.36–1.79)**		1.19 (0.90–1.56)
**Residence**			0.424		0.424	
Rural	1647 (60.6)	2884 (62.6)		1		
Urban	1069 (39.4)	1725 (37.4)		0.92 (0.76–1.13)		
**Region**			< 0.001		< 0.001	
Western	717 (26.4)	761 (16.5)		1		1
Southern	495 (18.2)	997 (21.6)		**1.90 (1.39–2.59)**	< 0.001	**2.46 (1.76–3.43)**
Northwestern	593 (21.8)	787 (17.1)		1.25 (0.92–1.71)	0.160	**1.56 (1.11–2.19)**
Northern	481 (17.7)	953 (20.7)		**1.87 (1.32–2.64)**	< 0.001	**2.25 (1.58–3.21)**
Eastern	431 (15.9)	1111 (24.1)		**2.43 (1.77–3.34)**	< 0.001	**2.89 (2.06–4.05)**
**Religion**			0.874		0.874	
Islam	2134 (78.6)	3632 (78.8)		1		
Christianity and others	582 (21.4)	978 (21.2)		0.99 (0.84–1.16)		
**Sex household head**			0.193		0.192	
Male	2015 (74.2)	3505 (76.0)		1		1
Female	702 (25.8)	1104 (24.0)		0.90 (078–1.05)		0.91 (0.79–1.06)
**Household Size**			0.378		0.378	
7 and above	1257 (46.3)	2062 (44.7)		1		
Less than 7	1460 (53.7)	2547 (55.3)		1.06 (0.93–1.22)		
**Working status**			0.864		0.864	
Not working	628 (23.1)	1054 (22.9)		1		
Working	2089 (76.9)	3555 (77.1)		1.01 (0.87–1.19)		
**Marital status**			0.891		0.891	
Not married	490 (18.0)	839 (18.2)		1		
Married	2227 (82.0)	3770 (81.8)		0.99 (0.84–1.17)		
**Education Level**			0.028		0.029	
No Education	1501 (55.2)	2357 (51.1)		1		1
Primary Education	376 (13.8)	657 (14.3)		1.11 (0.93–1.33)	0.250	1.04 (0.86–1.27)
Secondary Education	781 (28.8)	1433 (31.1)		1.17 (0.97–1.41)	0.100	1.10 (0.90–1.34)
Tertiary	59 (2.2)	162 (3.5)		**1.75 (1.17–2.61)**	0.006	**1.75 (1.16–2.64)**
**Wealth Index**			0.036		0.012	
Poorest	634 (34.4)	953 (20.7)		1		1
Poorer	539 (19.9)	1011 (21.9)		**1.25 (1.05–1.48)**	**0.011**	**1.29 (1.08–1.53)**
Middle	490 (18.0)	998 (21.6)		**1.36 (1.11–1.66)**	**0.003**	**1.41 (1.15–1.73)**
Richer	541 (19.9)	901 (19.5)		1.11 (0.88–1.41)	0.385	**1.42 (1.12–1.81)**
Richest	512 (18.8)	747 16.3)		0.97 (0.73–1.30)	0.852	**1.64 (1.20–2.24)**
**Parity**			0.179		0.203	
5 and above	517 (19.0)	806 (17.5)		1		1
2–4	1498 (55.2)	2516 (54.6)		1.08 (0.93–1.25)	0.310	1.05 (0.88–1.24)
1	702 (25.8)	1287 (27.9)		1.18 (0.98–1.41)	0.075	1.14 (0.92–1.40)
**Newspapers exposure**			0.537		0.539	
No	2575 (94.8)	4345 (94.3)		1		
Yes	141 (5.2)	264 (5.7)		1.11 (0.80–1.54)		
**Exposure to Radio**			0.016		0.016	
No	1646 (60.6)	2577 (55.9)		1		1
Yes	1071 (39.4)	2032 (44.1)		**1.21 (1.04–1.42)**		**1.24 (1.06–1.45)**
**Exposure to TV**			0.280		0.279	
No	2029 (74.7)	3550 (77.0)		1		
Yes	687 (25.3)	1060 (23.0)		0.88 (0.70–1.11)		
**Internet use**			0.661		0.661	
No	2432 (89.5)	4154 (90.1)		1		
Yes	285 (10.5)	455 (9.9)		0.94 (0.70–1.25)		
**Permission to access healthcare**			0.953		0.953	
Big problem	679 (25.0)	1147 (24.9)		1		
Not big problem	2038 (75.0)	3462 (75.1)		1.01 (0.84–12)		
**Distance to health facility**			0.918		0.918	
Big problem	1277 (47.0)	2177 (47.2)		1		
Not big problem	1440 (53.0)	2432 (52.8)		0.99 (0.82–1.19)		

^a^_=_ missing 112 (1.5%) respondents, **Bold**: significant at < 0.05. CoC is 62.9% (4609/7326: 95% CI:62.0–64.2)

## Discussion

This study focused on the level of CoC in Sierra Leone considering the utilization of three major aspects of maternal and newborn health such as eight ANC contacts, skilled birth attendance, maternal and neonatal postnatal care as well as their association with various socio-demographic and household related factors.

This study revealed that most women and their newborns did not receive maternal and newborn health services continuously. As per our results only 22% utilized 8 or more ANC contacts, 88% had skilled birth attendance while 90.7 and 90.4% of mothers and neonates utilized PNC respectively. However, only 17.9% of women in Sierra Leone utilized complete CoC for maternal and newborn health at all three levels. This low completion rate of CoC in the country means many women miss proven interventions at various contact points of the continuum hence risking delayed identification of maternal complications. Although the sensitivity analysis that used at least 4 ANC contacts showed a higher CoC completion rate, the primary results clearly showed that the new WHO eight ANC contacts model is yet to be institutionalized among women of childbearing age in Sierra Leone. Women who attended ANC after first trimester, from southern region, richer, used internet and had no big problem seeking permission were significantly associated with utilization of complete CoC for maternal and newborn health services at the three levels.

Another important observation in our study is the mismatch between high utilization of SBA and the persistently high record of maternal deaths. However, this has been shown in some other SSA countries. This mismatch could partly be attributed to delayed seeking of healthcare services for childbirth care and inadequate quality of care received [32–34]. Factors such as demotivated health workers due to poor remuneration, increased workload on health workers, limited availability of essential medical supplies and low quality pre-service and refresher training may partly explain the inadequate quality of care [29, 32, 33]. In Sierra Leone’s preservice training for SBAs produces; maternal and child health assistants (2 years), state enrolled community health nurses (two and half years), and state registered nurses (3 years) all of have the option of undertaking further midwifery training lasting for a maximum of 2 years [35, 36]. However, the quality of Sierra Leone’s preservice training is affected by; poor classroom attendance, demotivated facilitators due to delayed and low tutor allowances and poor infrastructure [36].

The magnitude of CoC is higher than those of studies conducted in a rural district of Lao People’s Democratic Republic [37], Uganda [20], Ghana [23] and Cambodia [38], as only 6.8, 10, 7.9 and 5.0% continued to receive CoC for maternal and newborn services at the three levels respectively. It is lower than those of studies conducted in Zambia [6], Egypt (50%) [39] and Pakistan (27%) [21]. These countries show that the disparities in utilization of complete continuum of care is because of shortages in human, financial and inadequate health system infrastructure, even though different ways of assessing continuum of care ware used. The low level in Sierra Leone could be explained by the impact of Ebola outbreak on reproductive health services among other socio-demographic factors [40].

Timing of ANC initiation was associated with utilization of complete CoC for maternal and newborn health services. Women who had their first ANC visit after the first trimester had lower odds of utilizing complete continuum of care compared to those who initiated ANC in their first trimester which similar finding was shown in Zambia [6]. A plausible explanation is that, the time at which first ANC visit is done has the utmost importance to ensure optimal health effects for both women and children [6]. This enables women to have more ANC contacts which offer an opportunity to establish baseline information on her general wellbeing, maternal and child counselling and education and creates good rapport with her health care provider hence increasing the chances of linking to the other components of continuum of care and promoting completion [30, 41]. Kinney et al. while analyzing data from sub-Saharan Africa showed that women who had initiated ANC from the first trimester had lower odds of maternal complications and mortality [42]. Therefore, there is need for stakeholders to promote timely ANC initiation as a way of ensuring increased CoC utilization as well as ensuring that quality ANC is provided to women. Although studies have linked maternal and newborn mortality reduction to the increased utilization and coverage of maternal healthcare services including ANC, field evidence shows that many countries especially those from SSA are having an issue of poor quality of services hence unable to guarantee effective, safe, people-centred, timely services recommended by WHO [43]. Therefore, as advocacy for programmes aimed at increasing utilization of maternal healthcare services in Sierra Leone is encouraged, ensuring that women receive quality maternal healthcare services should also be emphasized.

Women from the Southern region demonstrated a more likelihood of utilizing the complete continuum of care package compared to those from the Western region. Similarly, studies conducted in Ghana, Pakistan and Zambia documented the role of regional disparities in explaining the utilization of complete CoC [6, 21, 24]. Post-conflict in Sierra Leone, the health system has been described as fragile [40]. The Western region having a high concentration of skilled birth attendants with higher social amenities due to the high economic development compared to other regions [44, 45], one would expect higher CoC utilization. However, the Western region has increasingly registered a high number of urban poor and high standards of living which has led to inequitable access and affordability of social amenities and services including public and private health facilities, which negatively affects access to healthcare [46, 47]. In addition, the Western region being highly developed has led to health non-governmental organizations implementing maternal and child health programs to concentrate on other regions such as the Southern region could also partly explain this finding.

Women belonging to richer households were positively associated with complete continuum of maternal and newborn care, a finding consistent with several other studies including one from Cambodia [48] and another from [49]. The direct and indirect costs incurred when accessing care limit the ability of women from poor households to access maternal health services [50]. This implies that lower levels of wealth can also be a barrier to accessing maternal health services hence multi-sectoral actors and different attentions are needed to eliminate financial barriers to improve the continuity of maternity care in Sierra Leone.

Women who used internet were associated with higher odds of utilizing complete continuum of care compared to those who did not use. Similarly, a study conducted in an urban city in Nigeria suggests that internet is more popular compared to television and radio due to its high degree of interactivity, confidential and easily accessible especially on sensitive topics like reproductive health [51]. A review article revealed the important role the internet plays for women during pregnancy as it helps them to seek information in their early days of pregnancy and to make informed decisions [52]. The reasons behind the popularity of internet use in Sierra Leone could be that the internet provides various platforms to search information on maternal health as needed and equips the women with maternal health information on the importance of utilizing the continuum of care package [53].

Women who did not have a big problem in seeking permission to access a health facility were more likely to complete the three levels of continuum of care compared to those who had a big problem. High decision-making power has been shown to increase ANC contacts in Benin [54]. Increased decision-making power results from increased autonomy and empowerment among women, which depicts more access to health information and financial independence such that decisive decisions about her health are solely made by her [55].

### Strengths and limitations

The novelty of this study stems from the fact that, it is the first of its kind to have investigated continuum of care considering the current WHO guidelines in Sierra Leone. The study made use of cross-sectional survey data, which reflect the views of women aged 15–49 across the entire country and as such, the results and conclusions are based on a nationally representative survey. However, the study had some limitations. First, the data were collected based on women’s self-reports. This may lead to recall bias, information bias or social desirability bias. Second, the cross-sectional nature of the study does not permit causal inferences to be made. Lastly, the study included women who gave birth within 5 years preceding the survey (2015–2019) yet the WHO ANC guidelines were introduced in 2016 so considering the 8 ANC recommended contacts risks a lower prevalence of utilization of these ANC contacts.

## Conclusion

The low completion rate of the entire continuum of maternal care shown in this study indicates that women in Sierra Leone are not receiving the maximum possible health benefits from the existing health services. Stakeholders in maternal child health need to focus much on effective measures to ensure that the latest WHO ANC guidelines of at least 8 ANC contacts are implemented which low ANC utilization has negatively affected CoC completion. The predictors of completion of CoC have been demonstrated to operate at various levels - individual, household and community hence highlighting a need to contextualize efforts. Efforts to promote the use of maternal health services need to focus on regional differences in utilizing CoC, women with no access to internet, those with low autonomy, and those with low socio-economic status. Further research is needed in exploring and understanding why women do not complete the continuum of maternity care and for those who complete CoC, there is need to assess completion of CoC and quality of care received and maternal and neonatal outcomes. Besides, programs promoting maternal health utilization should emphasize accessing the full package rather than only specific components.

## Data Availability

The data set used is openly available upon permission from MEASURE DHS website (URL: https://www.dhsprogram.com/data/available-datasets.cfm). However, authors are not authorized to share this data set to the public but anyone interested in the data set can seek it with written permission from MEASURE DHS website (URL: https://www.dhsprogram.com/data/available-datasets.cfm).

## Abbreviations

EA: Enumeration area
AOR: Adjusted Odds Ratio
CI: Confidence Interval
COR: Crude Odds Ratio
DHS: Demographic Health Survey
SLDHS: Sierra Leone Demographic Health Survey
OR: Odds Ratio
SD: Standard Deviation
WHO: World Health Organization
ANC: Antenatal care
PNC: Postnatal care
SBA: Skilled Birth Attendance
CoC: Continuum of Care
SPSS: Statistical Package for Social Science
